# Epidemic and molecular characterization of fluoroquinolone-resistant *Shigella dysenteriae* 1 isolates from calves with diarrhea

**DOI:** 10.1186/s12866-020-02050-9

**Published:** 2021-01-06

**Authors:** Mingze Cao, Weiwei Wang, Liwei Zhang, Guanhui Liu, Xuzheng Zhou, Bing Li, Yuxiang Shi, Zhen Zhu, Jiyu Zhang

**Affiliations:** 1 Key Laboratory of New Animal Drug Project of Gansu Province, Key Laboratory of Veterinary Pharmaceutical Development of the Ministry of Agriculture, Lanzhou Institute of Husbandry and Pharmaceutical Sciences of CAAS, Jiangouyan, Qilihe District, Lanzhou, 730050 China; 2 College of Life Science and Food Engineering, Hebei University of Engineering, Hanshan District, Handan, 056038 China

**Keywords:** *Shigella dysenteriae*, Fluoroquinolone-resistant, QRDR, PMQR

## Abstract

**Background:**

The widespread distribution of antimicrobial-resistant *Shigella* has become a recurrent challenge in many parts of the developing world. Previous studies indicate that the host of *Shigella* has expanded from humans to animals. This study aimed to investigate the prevalence of fluoroquinolone resistance and associated molecular characterization of *S. dysenteriae* 1 isolated from calves.

**Results:**

All 38 unduplicated *S. dysenteriae* 1 isolates were collected from calves in Gansu Province from October 2014 to December 2016. According to MLST and PFGE analysis, these isolates were separated into 4 and 28 genotypes, respectively. The most common STs identified were ST228 (34.21%, 13/38) and ST229 (39.47%, 15/38), which were first found in the present study. All isolates harbored virulence genes, and the incidence of the seven virulence genes were *ipaH* (100%), *ipaBCD* (92.11%), *stx* (73.68%), *ial* (57.89%), *sen* (28.95%), *set1A* and *set1B* (0%). According to the results of antimicrobial susceptibilities, 76.32% (29/38) were resistant to fluoroquinolone and showed multidrug resistance. In a study on the polymorphism of quinolone resistance–determining region (QRDR) of *gyrA/B* and *parC/E* genes, we identified two mutations in *gyrA* (Ser83 → Leu and Asp87 → Asn) and *parC* (Ser80 → Ile and Ser83 → Leu), respectively. Among them, 55.17% (16/29) of resistant strains had the *gyrA* point mutations (Ser83 → Leu) and *parC* point mutation (Ser83 → Leu). Moreover, 41.38% (12/29) of isolates had all five point mutations of *gyrA* and *parC*. In addition, the prevalence of the plasmid-mediated quinolone resistance (PMQR) determinant genes was also investigated. All 29 fluoroquinolone-resistant isolates were positive for the *aac (6′)-Ib-cr* gene but negative for *qepA*, except for SD001. In addition, only 6 (20.69%, 6/29) isolates harbored the *qnr* gene, including two with *qnrB* (6.90%, 2/29) and four with *qnrS* (13.79%, 4/29).

**Conclusion:**

Given the increased common emergence of multidrug resistant isolates, uninterrupted surveillance will be necessary to understand the actual epidemic burden and control this infection.

**Supplementary Information:**

The online version contains supplementary material available at 10.1186/s12866-020-02050-9.

## Background

*Shigella* is one of the major pathogens that causes diarrheal diseases in humans and animals [1, 2]. Since the first highly toxigenic *Shigella dysenteriae* was isolated in 1898 [3], four species have been described in *Shigella* genus. Each subgroup can be divided into multiple subtypes according to biochemical and serological properties [4, 5]. Worldwide, there are approximately 164.7 million shigellosis infection cases in humans annually [5], while there are few data showing the prevalence in animals.

Antimicrobial therapy is the most effective way to fight shigellosis, so various kinds of drugs are widely used [6]. Fluoroquinolone, especially ciprofloxacin, was the first-line antibiotic for the treatment of shigellosis as recommended by the WHO [7]. However, antibiotic resistance usually occurs in the environment where antibiotics are frequently used [8, 9]. Furthermore, the accelerating accumulation and transmission of antimicrobial resistance genes (ARGs) among multidrug resistant (MDR) pathogens pose major difficulties in treating infections [10].

ARGs are widespread but cause problems only when present in pathogens [10]. Virulence genes have become significant marker of pathogenic bacteria. *S. dysenteriae* possesses diverse virulence genes, located on both the chromosome and/or the plasmid [11]. The common genetic loci are invasion plasmid antigen H (*ipaH*), invasion plasmid antigen genes (*ipaBCD)* and invasion associated locus (*ial*). In addition, the *Shigella* enterotoxin genes *set1A, set1B* (*ShET-1*), *sen* (*SHET-2*) and Shiga toxin gene *stx* were also reported to be responsible for initial watery diarrhea [12–14].

In this study, we aimed to investigate the epidemiology and molecular characteristics of *S. dysenteriae* isolates from calve farms in Gansu, China. Our analysis includes the antimicrobial resistance profiles, the molecular characterization of the mechanisms of resistance to fluoroquinolones, virulence gene profiles and the molecular characterization of *S. dysenteriae* isolates by pulsed-field gel electrophoresis (PFGE) and multilocus sequence typing (MLST).

## Results

### Bacterial isolation and identification

During our routine surveillance of bacillary dysentery, 38 *S. dysenteriae* were only found on six farms in Gansu Province. Detailed information on the study isolates is listed in Table S3. Among them, 20 isolates were isolated from 3 beef cattle farms, and 18 isolates were isolated from 3 dairy farms. There are 14 and 10 *S. dysenteriae* isolates from the same farms of Zhangye and Jinchang, respectively.

All 38 *S. dysenteriae* isolates were type 1 according to the results of the serotype reactions. In addition, based on the typical biochemical characteristics of *Shigella* spp., analysis of biochemical reactions indicated the presence of 3 biotypes (BTs) among these isolates (Tables 1 and S1). Among these BTs, BT2 (ability to ferment glucose, arabinose, and melibiose) was the predominant biotype, accounting for 86.84% (33/38). Furthermore, BT2 was widely isolated from each locus, with the exception of Baiyin.

**Table 1:** Biochemical characteristics of *S. dysentery 1* isolates

Biotype		Total (*n* = 38)	ZY (*n* = 14)	BY (*n* = 3)	LX (*n* = 3)	LZ (*n* = 5)	WW (*n* = 3)	JC (*n* = 10)
BT1	glucose+, mannose+, arabinose+, melibiose-	3 (7.89%)	0	3 (100%)	0	0	0	0
BT2	glucose+, mannose-, arabinose+, melibiose+	33 (86.84%)	14 (100%)	0	3 (100%)	5 (100%)	1 (33.33%)	10 (100%)
BT3	glucose+, mannose+, arabinose+, melibiose+	2 (5.26%)	0	0	0	0	2 (66.67%)	0

*ZY* Zhangye, *BY* Baiyin, *LX* Linxia, *LZ* Lanzhou, *WW* Wuwei, *JC* Jinchang

### MLST-based genotype analysis

**Fig. 1:**
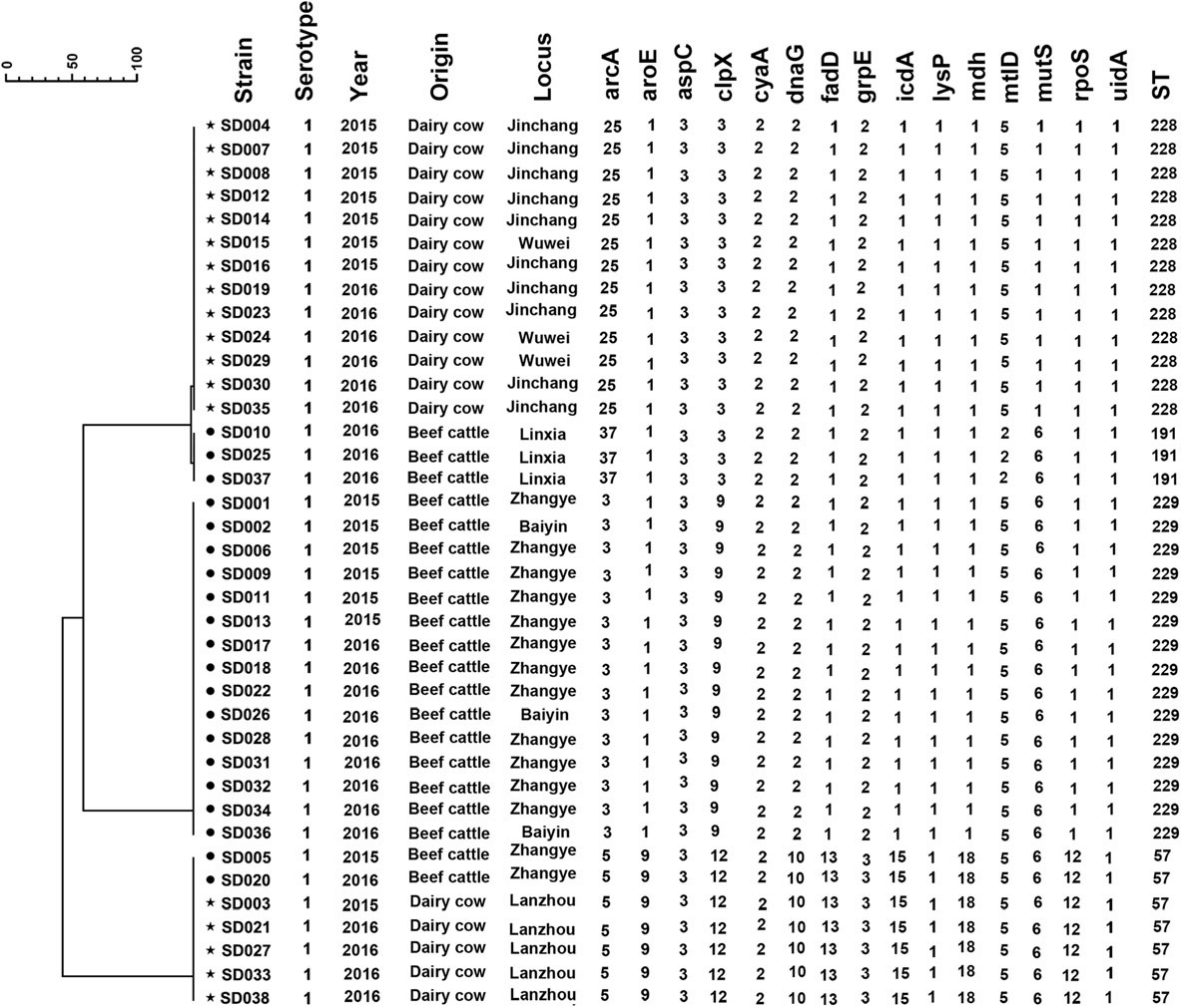
MLST clustering tree of *S. dysenteriae* isolates from 2014 to 2016 in diarrhea calves. The 38 isolates were analyzed using a 15 allele MLST as described in the Materials and Methods

**Fig. 2:**
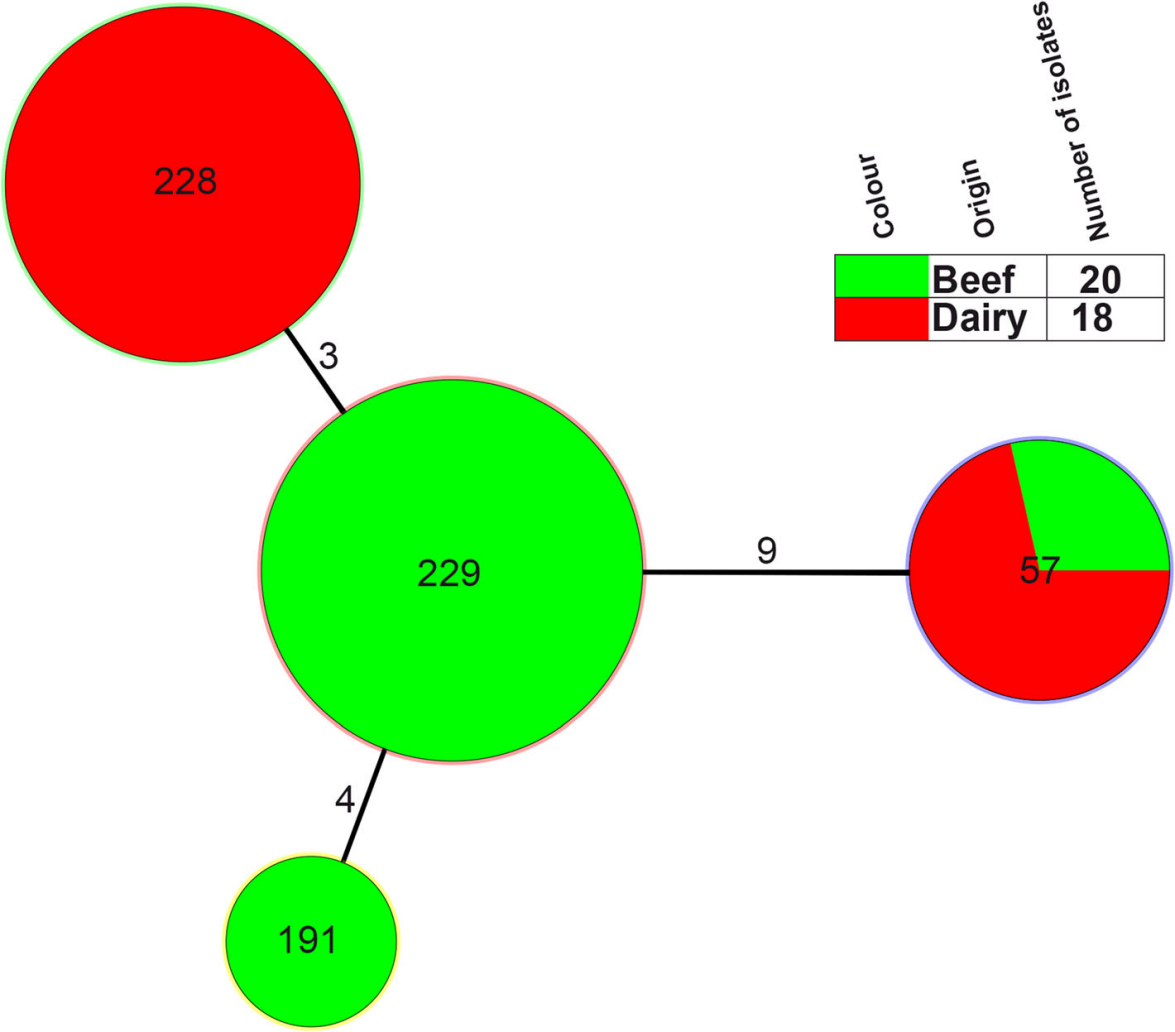
Minimum spanning tree of the isolates of 38 *S. dysenteriae* isolates from diarrhea calves based on multilocus sequence typing (MLST). The minimum spanning tree was constructed using the 4 identified STs obtained from the 38 isolates using BioNumerics Software. Each circle corresponds to a single ST. The shadow zones of circles’ filling in green correspond to beef, and red to dairy cows. The size of the circle is proportional to the number of isolates and the numbers over the branches means the difference between the two ST types. The corresponding color, number of isolates and background information are shown to the right of the minimum spanning tree. The number on the lines represent the number of different genes between the two conjoint STs

Thirty-eight *S. dysenteriae* isolates belonged to 4 MLST patterns (STs): ST57, ST191, ST228 and ST229. Among them, ST57 and ST191 were previously reported, while ST228 and ST229 were novel types. The allele number for each locus and the designation of the ST are listed in Fig. 1 and shared on the EcMLST website. The most common STs identified were ST228 (*n* = 13) and ST229 (*n* = 15), accounting for 73.68% (28/38). ST228 and ST229 were major ST types for dairy cows (Jinchang and Wuwei) and beef cattle (Zhangye and Baiyin) farms, respectively. In addition, these two ST types were different by 3 loci: *arcA*, *clpX*, *mutS*. In this study, each farm has a fixed ST type isolates, except Zhangye which contains ST229 and ST57 types (Figs. 1 and 2).

### PFGE-based genotype analysis

**Fig. 3:**
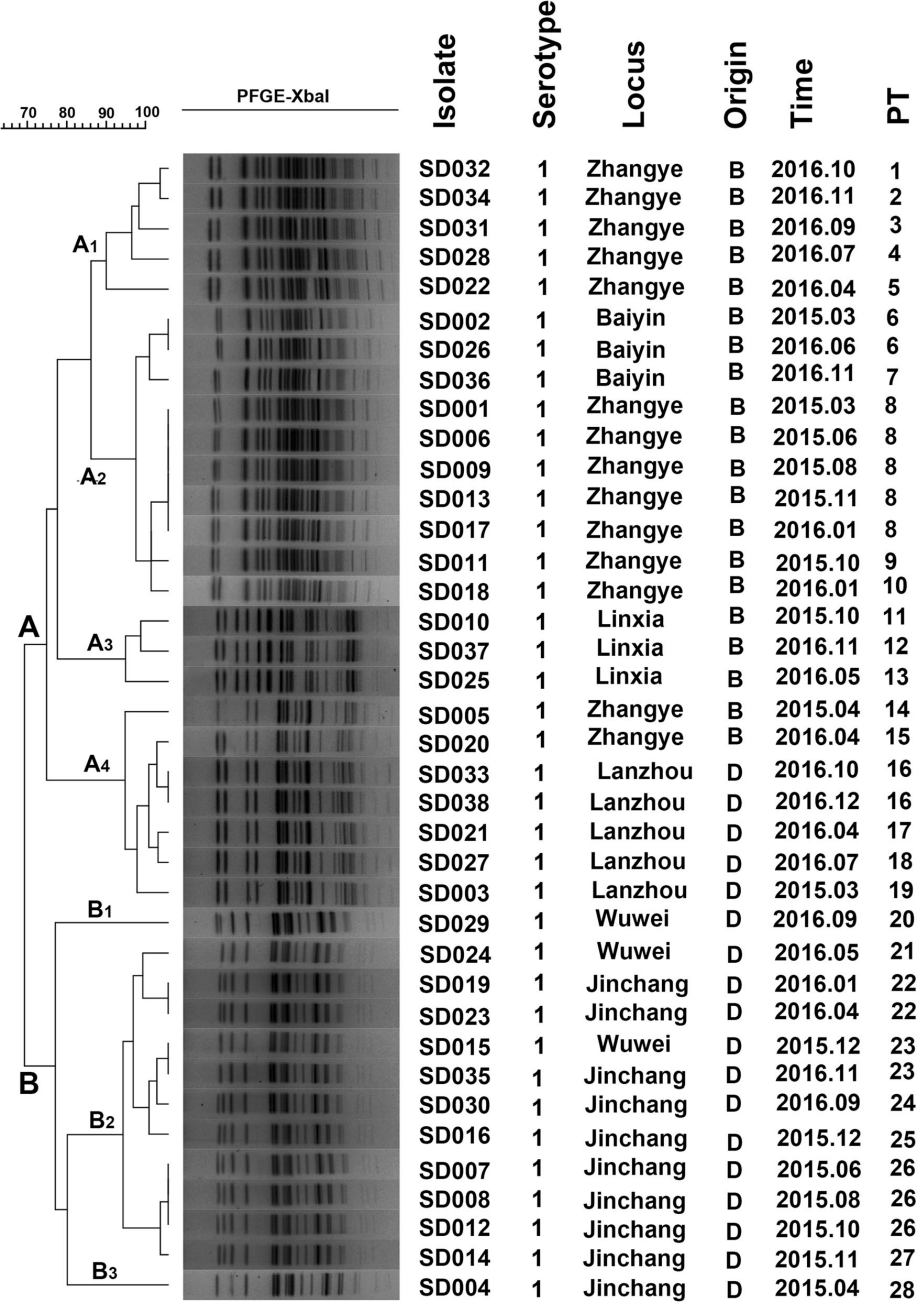
Dendrogram of 38 XbaI-digested *S. dysenteriae* isolates based on the cluster analysis of PFGE patterns. The dendrogram was constructed using the UPGMA clustering method. The PFGE pattern and background information for each strain are listed on the right side of the dendrogram

The genotypes and genetic relatedness of the 38 isolates were further determined by using PFGE. PFGE patterns of these *S. dysenteriae* isolates were heterogeneous; however, multiple PFGE patterns were present among these strains (Fig. 3). With approximately 80% similarity, XbaI-digested *S. dysenteriae* type 1 could be divided into 28 distinctive PFGE patterns (PT) and belonged to two major groups: A (A1-A4) and B (B1-B3), with 66% similarity. The cluster result of PFGE was similar with MLST, however, the same ST strain can be divided into several similar PT types. For example, the ST229 type is divided into 10 PT types, and the ST228 type is divided into 9 PT types. Interestingly, most strains in different geographical locations can be clustered individually, and strains in the farm can be divided into multiple PT types.

### Prevalence of virulence genes

A total of five virulence genes were detected in those isolates involving *ipaH*, *ipaBCD*, *ial*, *sen*, and *stx*. The most frequently observed virulence genes are *ipaH* (100%), *ipaBCD* (92.11%), *stx* (73.68%), and *ial* (57.89%). The *Shigella* enterotoxin genes *sen* (28.95%) are occasionally present in Wuwei and Jinchang isolates. None of the studied strains possessed the *set1A* or *set1B* gene.

Regarding the differences in virulence gene distributions, the 38 *S. dysenteriae* isolates fell into 5 gene profile types (VT) (Table 2). Among these VTs, VT IV (*n* = 17) and VT V (*n* = 11) were the most common, accounting for 44.34 and 28.95%, respectively. One interesting finding was the presence of the same and/or similar VTs in the same locus. In addition, 92.11% of isolates carried two or more virulence genes. Three Linxia isolates (7.89%) belonged to VT I, which was only positive for *ipaH*.

**Table 2:** Statistical the rate of each Virulence genes profile in *S. dysentery* serotype 1 isolates

VT	Virulence genes profiles	No. (%) isolates						
		Total (*n* = 38)	ZY (*n* = 14)	BY (*n* = 3)	LX (*n* = 3)	LZ (*n* = 5)	WW (*n* = 3)	JC (*n* = 10)
I	*ipaH* + *ipaBCD*-*ial*-*sen*-*stx*-	3 (7.89%)	0	0	3 (100%)	0	0	0
II	*ipaH* + *ipaBCD* + *ial*-*sen*-*stx*-	2 (5.26%)	0	2 (66.67%)	0	0	0	0
III	*ipaH* + *ipaBCD* + *ial* + *sen*-*stx*-	5 (13.16%)	2 (14.29%)	0	0	2 (40%)	1 (33.33%)	0
IV	*ipaH* + *ipaBCD* + *ial* + *sen*-*stx*+	17 (44.34%)	12 (85.71%)	1 (33.33%)	0	3 (60%)	0	1 (10%)
V	*ipaH* + *ipaBCD* + *ial*-*sen* + *stx*+	11 (28.95%)	0	0	0	0	2 (66.67%)	9 (90%)

*VT* Virulence genes profiles type, *ZY* Zhangye, *BY* Baiyin, *LX* Linxia, *LZ* Lanzhou, *WW* Wuwei, *JC* Jinchang

### Antimicrobial resistance profiles

The antimicrobial resistance profiles of the 20 antimicrobials for 38 *S. dysenteriae* are shown in Table 3. All *S. dysenteriae* isolates were uniformly multidrug resistant to at least 3 types of antimicrobial agents. Among them, resistance to E was the most common (36,94.74%), followed by AMP (35, 92.11%), KZ (33, 86.84%), CRO (32, 84.21%), CTX (32, 84.21%), TE (31, 81.58%), CN (31, 81.58%), ENR (26, 68.42%), LEV (25, 65.79%), CIP (25, 65.79%), NOR (15, 39.47%), OFX (15, 39.47%), C (15, 39.47%), and S (15, 39.47%). Fortunately, all 38 isolates were sensitive to AMC, FOX, FEP, MEM, IPM and AK. However, the resistance rate of fluoroquinolone antibiotics has reached 39.47 to 68.42%, which will narrow the choice of antibiotics.

**Table 3:** Statistical and analysis the result of antimicrobial susceptibility to 20 antibiotics for 38 *S. dysentery*

Antimicrobial			Antimicrobial resistance rate No. (%)						
			Total (*n* = 38)	Beef farm(*n* = 20)			Dairy farm(*n* = 18)		
				Zhangye(*n* = 14)	Baiyin(*n* = 3)	Linxia(*n* = 3)	Jinchang(*n* = 10)	Lanzhou(*n* = 5)	Wuwei(*n* = 3)
Fluoroquinolone	Norfloxacin	NOR	15(39.47%)	12(85.71%)	3(100%)	0	0	0	0
	Enrofloxacin	ENR	26(68.42%)	13(92.86%)	0	0	10(100%)	0	3(100%)
	Levofloxacin	LEV	25(65.79%)	12(85.71%)	0	0	10(100%)	0	3(100%)
	Ciprofloxacin	CIP	25(65.79%)	12(85.71%)	0	0	10(100%)	0	3(100%)
	Ofloxacin	OFX	15(39.47%)	12(85.71%)	3(100%)	0	0	0	0
β-lactam	Ampicillin	AMP	35(92.11%)	14(100%)	3(100%)	0	10(100%)	5(100%)	3(100%)
	Amoxycillin/Clavulanic acid	AMC	0	0	0	0	0	0	0
	Cephazolin	KZ	33(86.84%)	14(100%)	3(100%)	0	9(90%)	5(100%)	2(66.67%)
	Cefoxitin	FOX	0	0	0	0	0	0	0
	Ceftriaxone	CRO	32(84.21%)	14(100%)	0	0	10(100%)	5(100%)	3(100%)
	Cefotaxime	CTX	32(84.21%)	14(100%)	0	0	10(100%)	5(100%)	3(100%)
	Cefepime	FEP	0	0	0	0	0	0	0
	Meropenem	MEM	0	0	0	0	0	0	0
	Imipenem	IPM	0	0	0	0	0	0	0
Macrolides	Erythromycin	E	36(94.74%)	12(85.71%)	3(100%)	3(100%)	10(100%)	5(100%)	3(100%)
Chloramphenicol	Chloramphenicol	C	15(39.47%)	12(85.71%)	3(100%)	0	0	0	0
Tetracyclines	Tetracycline	TE	31(81.58%)	12(85.71%)	3(100%)	3(100%)	10(100%)	0	3(100%)
Aminoglycosides	Streptomycin	S	15(39.47%)	12(85.71%)	3(100%)	0	0	0	0
	Gentamicin	CN	31(81.58%)	12(85.71%)	3(100%)	3(100%)	10(100%)	0	3(100%)
	Amikacin	AK	0	0	0	0	0	0	0

Moreover, most of the isolates (29/38, 76.32%) were resistant to fluoroquinolone antibiotics. And 4 isolates were resistant to other fluoroquinolones other than CIP, including the resistance of SD020 to ENR and the resistance of SD002, SD026, SD036 to NOR and OFX. These resistant isolates were divided into 5 antimicrobial-resistance profile types (RT) (Table 4). Among these RTs, RT 4 (*n* = 11) and RT 5 (*n* = 12) were detected easily, accounting for 37.93 and 41.38%, respectively. All of the fluoroquinolone-resistant isolates were multidrug resistant (MDR). To be specific, 41.8% (12/29) of fluoroquinolone-resistant isolates were resistant to each class of antibiotics. Regarding the test for the virulence genes of *ipaBCD, sen, stx*, there are statistically significant difference between RT type and fluoroquinolone sensitive strains (P<0.05) (Table 5).

**Table 4:** Antimicrobial profile and amino acid types in QRDRs and PMQRs analysis of *S. dysentery* with resistance to fluoroquinolones

Strain name	Antimicrobial profile		QRDRs				PMQR			ST	PT
			*gyrA*		*parC*		*aac(6*′*)-Ib-cr*	*qnr*	*qepA*		
			83 (S)	87 (D)	80 (S)	83 (S)					
SD020	RT1	ENR/KZ/CRO/CTX	L	D	I	L	+	–	–	57	15
SD002	RT2	NOR/OFX/KZ/E/C/TE/S/CN	L	D	S	L	+	–	–	229	6
SD026	RT2	NOR/OFX/KZ/E/C/TE/S/CN	L	D	S	L	+	–	–	229	6
SD036	RT2	NOR/OFX/KZ/E/C/TE/S/CN	L	D	S	L	+	–	–	229	7
SD015	RT3	ENR/LEV/CIP/ CRO/CTX/E/TE/CN	L	D	S	L	+	–	–	228	23
SD016	RT3	ENR/LEV/CIP/ CRO/CTX/E/TE/CN	L	D	S	L	+	–	–	228	25
SD004	RT4	ENR/LEV/CIP/ KZ/CRO/CTX/E/TE/CN	L	D	S	L	+	–	–	228	28
SD007	RT4	ENR/LEV/CIP/ KZ/CRO/CTX/E/TE/CN	L	D	S	L	+	–	–	228	26
SD008	RT4	ENR/LEV/CIP/ KZ/CRO/CTX/E/TE/CN	L	D	S	L	+	*qnrB*	–	228	26
SD012	RT4	ENR/LEV/CIP/ KZ/CRO/CTX/E/TE/CN	L	D	S	L	+	–	–	228	26
SD014	RT4	ENR/LEV/CIP/ KZ/CRO/CTX/E/TE/CN	L	D	S	L	+	–	–	228	27
SD019	RT4	ENR/LEV/CIP/ KZ/CRO/CTX/E/TE/CN	L	D	S	L	+	–	–	228	22
SD023	RT4	ENR/LEV/CIP/ KZ/CRO/CTX/E/TE/CN	L	D	S	L	+	*qnrB*	–	228	22
SD024	RT4	ENR/LEV/CIP/ KZ/CRO/CTX/E/TE/CN	L	D	S	L	+	–	–	228	21
SD029	RT4	ENR/LEV/CIP/ KZ/CRO/CTX/E/TE/CN	L	D	S	L	+	–	–	228	20
SD030	RT4	ENR/LEV/CIP/ KZ/CRO/CTX/E/TE/CN	L	D	S	L	+	–	–	228	24
SD035	RT4	ENR/LEV/CIP/ KZ/CRO/CTX/E/TE/CN	L	D	S	L	+	–	–	228	23
SD001	RT5	NOR/ENR/LEV/CIP/OFX/ KZ/CRO/CTX/E/C/TE/S/CN	L	N	I	L	+	–	+	229	8
SD006	RT5	NOR/ENR/LEV/CIP/OFX/ KZ/CRO/CTX/E/C/TE/S/CN	L	N	I	L	+	–	–	229	8
SD009	RT5	NOR/ENR/LEV/CIP/OFX/ KZ/CRO/CTX/E/C/TE/S/CN	L	N	I	L	+	–	–	229	8
SD011	RT5	NOR/ENR/LEV/CIP/OFX/ KZ/CRO/CTX/E/C/TE/S/CN	L	N	I	L	+	–	–	229	9
SD013	RT5	NOR/ENR/LEV/CIP/OFX/ KZ/CRO/CTX/E/C/TE/S/CN	L	N	I	L	+	–	–	229	8
SD017	RT5	NOR/ENR/LEV/CIP/OFX/ KZ/CRO/CTX/E/C/TE/S/CN	L	N	I	L	+	–	–	229	8
SD018	RT5	NOR/ENR/LEV/CIP/OFX/ KZ/CRO/CTX/E/C/TE/S/CN	L	N	I	L	+	–	–	229	10
SD022	RT5	NOR/ENR/LEV/CIP/OFX/ KZ/CRO/CTX/E/C/TE/S/CN	L	N	I	L	+	*qnrS*	–	229	6
SD028	RT5	NOR/ENR/LEV/CIP/OFX/ KZ/CRO/CTX/E/C/TE/S/CN	L	N	I	L	+	*qnrS*	–	229	4
SD031	RT5	NOR/ENR/LEV/CIP/OFX/ KZ/CRO/CTX/E/C/TE/S/CN	L	N	I	L	+	*qnrS*	–	229	3
SD032	RT5	NOR/ENR/LEV/CIP/OFX/ KZ/CRO/CTX/E/C/TE/S/CN	L	N	I	L	+	*qnrS*	–	229	1
SD034	RT5	NOR/ENR/LEV/CIP/OFX/ KZ/CRO/CTX/E/C/TE/S/CN	L	N	I	L	+	*qnrS*	–	229	2

**Table 5:** Prevalence of virulence genes in the different antimicrobial-resistance profile types strains

Gene	Total (*n* = 38)	RT1 (*n* = 1)	RT2 (*n* = 3)	RT3 (*n* = 2)	RT4 (*n* = 11)	RT5 (*n* = 12)	Other (*n* = 9)	*P*-value
*ipaH*	38 (100%)	1 (100%)	3 (100%)	2 (100%)	11 (100%)	12 (100%)	9 (100%)	–
*ipaBCD*	35 (92.11%)	1 (100%)	3 (100%)	2 (100%)	11 (100%)	12 (100%)	6 (66.67%)	0.001
*ial*	22 (57.89%)	1 (100%)	1 (33.33%)	0 (0%)	2 (18.18%)	12 (100%)	6 (66.67%)	0.542
*sen*	11 (28.95%)	0 (0%)	0 (0%)	2 (100%)	9 (81.82%)	0 (0%)	0 (0%)	0.028
*stx*	28 (73.68%)	0 (0%)	1 (33.33%)	2 (100%)	10 (90.91%)	12 (100%)	3 (33.33%)	0.002

Other: fluoroquinolone sensitive strains

In addition, the resistance profiles of strains which isolated from the same farms were very similar. And the isolates belonging to ST229 (A1 and A2 groups) and ST228 (B group) were resistant to fluoroquinolone antibiotics, while the isolates belonging to ST 57 (A4) and ST191 (A3) were sensitive to those antibiotics, except for SD020 isolated from Zhangye area with ST57 (PT15).

### ARG analysis for fluoroquinolone-resistant *S. dysenteriae* isolates

To determine the molecular characterization in fluoroquinolone-resistant *S. dysenteriae*, both SNPs in QRDR of *gyrA*/*B* and *parC*/*E* genes and PMQR genes were analyzed. In 29 fluoroquinolone-resistant isolates, there was no strain that displayed mutations in the *gyrB* and *parE* genes, although some mutations in *gyrA* and *parC* were identified in each resistant isolate (Table 4). All resistant isolates in the present study carried common mutations in *gyrA* codon 83 (S → L) and *parC* codon 83 (S → L). Furthermore, the isolates with ST228 only carried the mutations in *gyrA* codon 83 (S → L) and *parC* codon 83 (S → L). While most of the isolates with ST229 carried all of the four mutated locuses, except isolates from Baiyin.

The PMQR genes *qnr*, *aac (6′)-Ib-cr*, and *qepA* occur worldwide and are increasingly detected in clinical isolates of Enterobacteriaceae [15, 16]. In our study, *aac (6′)-Ib-cr* was the most common PMQR gene and harbored by each isolate; however, all isolates were negative for *qepA*, except SD001. Only seven (7/29, 24.14%) isolates harbored the *qnr* gene, and no isolate harbored *qnr*, *aac (6′)-Ib-cr*, and *qepA* simultaneously. Interestingly, the *qnrB* (*n* = 2) gene and *qnrS* (*n* = 5) were belonged into ST228 and ST229, respectively.

## Discussion

Invasive diarrhea due to *Shigella* species remains an important public health problem in developing countries [17]. All four species of *Shigella* can cause shigellosis, but *S. flexneri* and *S. sonnei* are the most prevalent [18]. Present study confirmed the existence of diverse *Shigella* isolates in calves in local epidemiological studies. A total of 136 *Shigella* isolates were collected in this study, including 54 *S. flexneri* (1.63%, 54/3321), 44 *S. sonnei* (1.32%, 44/3321), and 38 *S. dysenteriae* (1.14%, 38/3321), and no *S. boydii* was found.

Strain molecular characterization is important for epidemiological studies. MLST, PFGE and MLVA techniques were widely used in laboratory for Shigella study, unfortunately, we don’t have a widely recognized MLVA methods to study *S. dysentery*. The next generation sequencing is the reference technique today, however, it requires professional analytical methods and tedious operations, making it unable to be widely carried out in all laboratories compared with MLST and PFGE.

In our study, the majority of isolates (*n* = 13 and *n* = 15) belonged to new ST types ST228 and ST229. However, ST148, ST252, and ST1739 were previously reported in human isolates [19]. PFGE is the most common typing procedure currently used with *Shigella* spp. because of its high discriminatory power [20]. Taking PFGE as a reference epidemiological tool, the clustering of these diverse PTs allows us to learn more about the epidemiological characteristics of *S. dysenteriae* in specific geographical regions.

The ability of *Shigella* spp. to cause shigellosis is attributed to the expression of arrays of virulence genes associated with colonization, invasion/penetration and toxin-mediated disease [21]. Virulence genes responsible for the pathogenesis of shigellosis are often multifactorial and coordinately regulated [22]. Estimating the existence of virulence determinants in *Shigella* would help us better understand its pathogenicity [21]. *IpaH* takes responsibility for the strain spread from cell to cell and the modification of host response to infection [23]. All *Shigella* spp. were positive for *ipaH* as a typical marker because this gene exists in multiple copies on both the chromosome and the invasion plasmid. Additionally, in this study, most isolates simultaneously harbored the *stx*, *ial* and *sen* genes. The diversity of the observed virulence genes results in dysentery in hosts. Notably, these virulence genes are indistinguishable from human *Shigella*.

For shigellosis, antibiotic therapy can reduce the duration and severity of the illness [24]. Based on previous reports, fluoroquinolones and third-generation cephalosporins are the best choice for empiric treatment for severe bacterial diarrheas caused by *S. dysenteriae* [25]. However, resistance will emerge to any antimicrobial agent used intensively, and the differential selection pressures of antimicrobials lead to differences in drug resistance [26]. These factors have contributed to *Shigella* strains acquiring resistance to the latest drug of choice, together with other effective drugs after therapy shigellosis. In addition, the resistance rate of *S. dysenteriae* 1 isolates to fluoroquinolones has achieved at an alarming rate, and the isolates have demonstrated multidrug resistance. Our current study indicated that continuous surveillance of the resistance pattern in pathogens would be essential for the choice of appropriate antimicrobial therapy and control the spread of *Shigella*.

The most common mechanism of the highly level resistance to quinolone has been mostly attributed to accumulation of sequential mutations in DNA gyrase and DNA topoisomerase IV, typically of *gyrA* at codon 83 and/or 87, and of *par*C at codon 80 [27, 28]. In addition, different mutation loci and different mutations at the same locus may result in different quinolone susceptibility levels [29, 30]. To date, limited studies have examined quinolone resistance mechanisms among *Shigella* isolates in animals. In the current study, we successfully investigated fluoroquinolone resistance-conferring mutations by sequencing QRDR of *S. dysenteriae* 1 isolated in different geographical locations. According to our results, only two main combinations of mutations were observed: *gyrA* codons 83 (Ser → Leu), and *parC* codons 83 (Ser → Leu) (55.17%, 16/29); *gyrA* codons 83 (Ser → Leu), 87 (Asp→Asn) and *parC* codons 80 (Ser → Ile), 83 (Ser → Leu) (41.38%, 12/29). This phenomenon of high resistance rate and single mutation pattern is likely related to the large-scale use of single quinolone antibiotics by veterinarians.

Generally, the PMQR determinants are located on mobile genetic elements, which may be associated with mobile or transposable elements among members of the Enterobacteriaceae family [31]. Over the past few years, the dissemination of PMQR genes among quinolone-resistant *Shigella* isolates has been surveyed in some studies and has emerged as an important issue across the world [32–35]. It has been reported that *aac (6′)-Ib-cr* significantly increases the frequency of selection of chromosomal mutants to reduce ciprofloxacin activity by N-acetylation at the amino nitrogen on its piperazinyl substituent [36]. The *aac (6′)-Ib-cr*–positive *Shigella* was first isolated in 1998 [37]; however, the most striking finding of our previous and present study was the *aac (6*′*)-Ib-cr* widely present in all quinolone-resistant isolates [38, 39]. The *qnr* family could protect DNA gyrase against quinolones and confer low-level resistance to nalidixic acid and reduced susceptibility to fluoroquinolone, especially to ciprofloxacin [40]. The *qnr* gene has occurred worldwide and contains a variety of subtypes, but it was not a common epidemic genotype according to our study. Otherwise, the plasmid-mediated *qepA* gene can encode a 14-transmembrane segment efflux pump and extrude several quinolones from bacteria, thereby conferring low levels of resistance to these antimicrobial agents and favoring the selection of new alterations that are able to induce full quinolone resistance [41, 42]. Compared with other PMQR determinants, *qepA* is a new resistance gene, first described in 2007 [43], so it is not difficult to understand the lowest frequencies.

The QRDR mutation and PMQR determinants are both important to quinolone resistance. Compared with mutations in the QRDR, PMQR could not be the direct reason for quinolone and fluoroquinolone resistance [44]. However, the presence of PMQR genes may facilitate the selection of QRDR mutations that result in higher levels of quinolone resistance.

## Conclusion

In this study described fluoroquinolone resistance and virulence genes among *S. dysenteriae* strains isolated from calves and the molecular characterization involved. To systematically understand *S. dysenteriae*, PFGE and MLST methods were applied to genetically characterize the 38 isolates. PFGE based on XbaI digestion divided the 38 isolates into 28 PTs, while MLST based on 15 housekeeping genes differentiated the 38 isolates into 4 STs. Although MLST provided suitable discrimination in *S. dysenteriae* subtyping, PFGE might exhibit a higher discriminatory ability. Overall, the data from this study will provide a useful typing resource, which will provide a scientific basis for addressing clinical and epidemiological issues regarding *S. dysenteriae*. Given this knowledge, continuous and extensive surveillance will be essential to explore and prevent the spread of the epidemic.

## Methods

### Bacterial strains and serotyping

From October 2014 to December 2016, we tracked the calf diarrhea caused by *Shigella* from 26 farms in northwestern regions of China. A total of 3321 fresh stool samples from calves with diarrhea (3 to 20 days) were collected for detecting *Shigella*. All of the isolates were collected directly from fresh stool samples following plating on *Salmonella-Shigella* (*SS*) selective agar and confirming on MacConkey (*MAC*) agar with 37 °C for 24 h. The colorless, semitransparent, smooth, and moist circular plaques were considered presumptive *Shigella* for further 16S rDNA (TaKaRa, Japan) and biochemical confirmation.

The biochemical reactions of the presumptive positive *Shigella* isolates were tested by API20E kits (bioMerieux, France), and the serotype was tested by a commercially available agglutinating antibody kit (Denka Seiken, Tokyo, Japan) according to the manufacturers’ recommendations.

### Multilocus sequence typing (MLST)

All isolates were subjected to MLST according to the protocols described in the EcMLST database (http://www.shigatox.net/ecmlst). PCR amplification conditions of 15 housekeeping genes were as follows: 95 °C for 5 min; 30 cycles of 94 °C for 30 s, 55 °C for 90 s, and 72 °C for 1 min; and 72 °C for 5 min with ExTaq DNA polymerase (Takara, Dalian, China). The PCR products were sequenced bidirectionally, and sequences of the 15 housekeeping genes were edited by using SeqMan 7.0. Then, these sequences were uploaded to the EcMLST website for comparison, which allowed us to determine the gene and ST type [45]. Clustering and minimum spanning tree (MST) analysis were used to infer relationships among the isolates using the fingerprint analysis software BioNumerics (version 7.1).

### Pulsed-field gel electrophoresis (PFGE)

DNA fingerprinting was performed by PFGE. Intact agarose-embedded chromosomal DNA of *S. dysenteriae* isolates was digested with restriction enzyme XbaI (TaKaRa, Japan), and restriction fragments were separated on the CHEF Mapper XA system (Bio-Rad) in 1% SeaKem Gold agarose gel (Lonza, USA) in 0.5x TBE buffer with a size range of 30–700 kb. Electrophoretic parameters were determined by multiple screening for the most suitable value, which included switching times from 2.16 to 54.17 s, voltage 6 v/cm, angle 120° and run time 21 h. *Salmonella enterica serotype Braenderup* strain H9812 was used as a molecular size marker for this analysis.

The gel image was captured by Universal Hood II (Bio-RAD, USA), and the PFGE profiles were analyzed by BioNumerics (version 7.1). Degrees of homology were determined by comparing the bands, and the clustering tree indicating relative genetic similarity was constructed by the unweighted pair group method with arithmetic averages (UPGMA).

### Detection of virulence genes

All *S. dysenteriae* isolates were examined for the presence of seven virulence genes, invasion plasmid antigen H (*ipaH*), invasion plasmid antigen genes (*ipaBCD*), invasion associated locus (*ial*), Shiga toxin gene (*stx*), and *Shigella* enterotoxin genes (*set1A*, *set1B*, and *sen*) by PCR according to published procedures [13, 27]. The primers for these virulence genes are listed in Table S2. Amplification products were separated by 1% agarose gel electrophoresis and stained with ethidium bromide.

### Determination of antimicrobial susceptibility

All of the isolated strains were tested for their antimicrobial susceptibility against a variety of 20 antibiotic discs (Oxoid, UK) on Mueller–Hinton agar following the guidelines of the Clinical and Laboratory Standards Institute (CLSI) [46]. The antimicrobial discs used in this study were norfloxacin (NOR, 10 μg), enrofloxacin (ENR, 5 μg), levofloxacin (LEV, 5 μg), ciprofloxacin (CIP, 5 μg), ofloxacin (OFX, 5 μg), ampicillin (AMP, 10 μg), amoxycillin/clavulanic acid (AMC, 30 μg), cephazolin (KZ, 30 μg), cefoxitin (FOX, 30 μg), ceftriaxone (CRO, 30 μg), cefotaxime (CTX, 30 μg), cefepime (FEP, 30 μg), imipenem (IPM, 10 μg), meropenem (MEM, 10 μg), erythromycin (E, 15 μg), chloramphenicol (C, 30 μg), tetracycline (TE, 30 μg), streptomycin (S, 10 μg), gentamicin (CN, 10 μg), and amikacin (AK, 30 μg). *E. coli* ATCC 25922 was used for quality control organisms according to the CLSI standard.

### Detection of fluoroquinolone resistance genes

To understand the underlying mechanism of fluoroquinolone resistance, four QRDR genes, DNA gyrase (*gyrA* and *gyrB*) and topoisomerase IV (*parC* and *parE*), and six PMQR determinant genes, *qnrA*, *qnrB*, *qnrD*, *qnrS*, *aac (6′)-Ib-cr* and qepA, were amplified for fluoroquinolone-resistant isolates in our study [4, 47, 48]. The obtained PCR fragments were sequenced after purification and analyzed through comparison with sequences in GenBank. The primer information is listed in Table S3.

### Statistical analysis

The *P*-value in each gene of prevalence rates across RT and Other were calculated using Chi-square test (SPSS 19.0 version, IBM, New York, USA) to find any relationship. A P<0.05 was statistically significant.

## Supplementary Information

**Additional file 1: Table S1.** Strain information of *S. dysenteriae* isolates from diarrheal calves in this study.

**Additional file 2: Table S2.** Primers for the detection of virulence genes.

**Additional file 3: Table S3.** Primers for the detection of fluoroquinolone resistance–determining genes.

## Data Availability

The data supporting the findings of this study are contained within the manuscript.

## Abbreviations

PFGE: Pulsed-field gel electrophoresis
MLST: Multilocus sequence typing
ARGs: Antimicrobial resistance genes
MDR: Multidrug resistant
BTs: Biotypes
VT: Virulence profile types
NOR: Norfloxacin
ENR: Enrofloxacin
LEV: Levofloxacin
CIP: Ciprofloxacin
OFX: Ofloxacin
AMP: Ampicillin
AMC: Amoxycillin/Clavulanic
KZ: Cephazolin
FOX: Cefoxitin
CRO: Ceftriaxone
CTX: Cefotaxime
FEP: Cefepime
MEM: Meropenem
IPM: Imipenem
E: Erythromycin
C: Chloramphenicol
TE: Tetracycline
S: Streptomycin
CN: Gentamicin
AK: Amikacin
