# Supplementary material for: Epidemic and molecular characterization of fluoroquinolone-resistant Shigella dysenteriae 1 isolates from calves with diarrhea
Source: BMC Microbiol. 2021 Jan 6;21:6. doi: 10.1186/s12866-020-02050-9 (PMC7789508; doi:10.1186/s12866-020-02050-9)
Supplement: Supplementary file 1 — Additional file 1: Table S1. Strain information of S. dysenteriae isolates from diarrheal calves in this study. [file 12866_2020_2050_MOESM1_ESM.docx]

Table S1 Strain information of *S. dysentery* isolates from diarrheal calves in this study.

| Strain name | Serotype | Origin | Locus | Date | Biotype |
| --- | --- | --- | --- | --- | --- |
| SD001 | 1 | Beef cattle | Zhangye | 2015.03 | BT2 |
| SD002 | 1 | Beef cattle | Baiyin | 2015.03 | BT1 |
| SD003 | 1 | Dairy cow | Lanzhou | 2015.03 | BT2 |
| SD004 | 1 | Dairy cow | Jinchang | 2015.04 | BT2 |
| SD005 | 1 | Beef cattle | Zhangye | 2015.04 | BT2 |
| SD006 | 1 | Beef cattle | Zhangye | 2015.06 | BT2 |
| SD007 | 1 | Dairy cow | Jinchang | 2015.06 | BT2 |
| SD008 | 1 | Dairy cow | Jinchang | 2015.08 | BT2 |
| SD009 | 1 | Beef cattle | Zhangye | 2015.08 | BT2 |
| SD010 | 1 | Beef cattle | Linxia | 2015.10 | BT2 |
| SD011 | 1 | Beef cattle | Zhangye | 2015.10 | BT2 |
| SD012 | 1 | Dairy cow | Jinchang | 2015.10 | BT2 |
| SD013 | 1 | Beef cattle | Zhangye | 2015.11 | BT2 |
| SD014 | 1 | Dairy cow | Jinchang | 2015.11 | BT2 |
| SD015 | 1 | Dairy cow | Wuwei | 2015.12 | BT2 |
| SD016 | 1 | Dairy cow | Jinchang | 2015.12 | BT2 |
| SD017 | 1 | Beef cattle | Zhangye | 2016.01 | BT2 |
| SD018 | 1 | Beef cattle | Zhangye | 2016.01 | BT2 |
| SD019 | 1 | Dairy cow | Jinchang | 2016.01 | BT2 |
| SD020 | 1 | Beef cattle | Zhangye | 2016.04 | BT2 |
| SD021 | 1 | Dairy cow | Lanzhou | 2016.04 | BT2 |
| SD022 | 1 | Beef cattle | Zhangye | 2016.04 | BT2 |
| SD023 | 1 | Dairy cow | Jinchang | 2016.04 | BT2 |
| SD024 | 1 | Dairy cow | Wuwei | 2016.05 | BT3 |
| SD025 | 1 | Beef cattle | Linxia | 2016.05 | BT2 |
| SD026 | 1 | Beef cattle | Baiyin | 2016.06 | BT1 |
| SD027 | 1 | Dairy cow | Lanzhou | 2016.07 | BT2 |
| SD028 | 1 | Beef cattle | Zhangye | 2016.07 | BT2 |
| SD029 | 1 | Dairy cow | Wuwei | 2016.09 | BT3 |
| SD030 | 1 | Dairy cow | Jinchang | 2016.09 | BT2 |
| SD031 | 1 | Beef cattle | Zhangye | 2016.09 | BT2 |
| SD032 | 1 | Beef cattle | Zhangye | 2016.10 | BT2 |
| SD033 | 1 | Dairy cow | Lanzhou | 2016.10 | BT2 |
| SD034 | 1 | Beef cattle | Zhangye | 2016.11 | BT2 |
| SD035 | 1 | Dairy cow | Jinchang | 2016.11 | BT2 |
| SD036 | 1 | Beef cattle | Baiyin | 2016.11 | BT1 |
| SD037 | 1 | Beef cattle | Linxia | 2016.11 | BT2 |
| SD038 | 1 | Dairy cow | Lanzhou | 2016.12 | BT2 |
