# Supplementary material for: Epidemic and molecular characterization of fluoroquinolone-resistant Shigella dysenteriae 1 isolates from calves with diarrhea
Source: BMC Microbiol. 2021 Jan 6;21:6. doi: 10.1186/s12866-020-02050-9 (PMC7789508; doi:10.1186/s12866-020-02050-9)
Supplement: Supplementary file 2 — Additional file 2: Table S2. Primers for the detection of virulence genes. [file 12866_2020_2050_MOESM2_ESM.docx]

Table S2 Primers for detection of virulence genes.

| Target | Primer sequence (5’ to 3’) | Amplicon size (bp) | Reference |
| --- | --- | --- | --- |
| *ipaH* | F: TGGAAAAACTCAGTGCCTCT | 423 | Das A, et al., 2016 |
|  | R: CCAGTCCGTAAATTCATTCT |  |  |
| *ipaBCD* | F: GCTATAGCAGTGACATG | 500 | Faruque SM, et al., 2002 |
|  | R: ACGAGTTCGAAGCACTC |  |  |
| *ial* | F: CTGGATGGTATGGTGAGG | 320 | Das A, et al., 2016 |
|  | R: GGAGGCCAACAATTATTTCC |  |  |
| *sen* | F: ATGTGCCTGCTATTATTTAT | 799 | Das A, et al., 2016 |
|  | R: CATAATAATAAGCGGTCAGC |  |  |
| *Set1A* | F: TCACGCTACCATCAAAGA | 309 | Das A, et al., 2016 |
|  | R: TATCCCCCTTTGGTGGTA |  |  |
| *Set1B* | F: GTGAACCTGCTGCCGATATC | 147 | Das A, et al., 2016 |
|  | R: ATTAGTGGATAAAAATGACG |  |  |
| *stx* | F: CAGTTAATGTGGTTGCGAAG | 895 | Vargas M, et al., 1999 |
|  | R: CTGCTAATAGTTCTGCGCATC |  |  |

**References**

1. Das, A., Natarajan, M., Mandal, J., 2016, The Emergence of Quinolone Resistant Shigella sonnei, Pondicherry, India. Plos One. 11, e160290.

2. [Faruque, SM](https://www.ncbi.nlm.nih.gov/pubmed/?term=Faruque%20SM%5BAuthor%5D&cauthor=true&cauthor_uid=12147489)., [Khan, R](https://www.ncbi.nlm.nih.gov/pubmed/?term=Khan%20R%5BAuthor%5D&cauthor=true&cauthor_uid=12147489)., [Kamruzzaman, M](https://www.ncbi.nlm.nih.gov/pubmed/?term=Kamruzzaman%20M%5BAuthor%5D&cauthor=true&cauthor_uid=12147489)., [Yamasaki, S](https://www.ncbi.nlm.nih.gov/pubmed/?term=Yamasaki%20S%5BAuthor%5D&cauthor=true&cauthor_uid=12147489)., [Ahmad, QS](https://www.ncbi.nlm.nih.gov/pubmed/?term=Ahmad%20QS%5BAuthor%5D&cauthor=true&cauthor_uid=12147489)., [Azim, T](https://www.ncbi.nlm.nih.gov/pubmed/?term=Azim%20T%5BAuthor%5D&cauthor=true&cauthor_uid=12147489)., [Nair, GB](https://www.ncbi.nlm.nih.gov/pubmed/?term=Nair%20GB%5BAuthor%5D&cauthor=true&cauthor_uid=12147489)., [Takeda, Y](https://www.ncbi.nlm.nih.gov/pubmed/?term=Takeda%20Y%5BAuthor%5D&cauthor=true&cauthor_uid=12147489)., [Sack, DA](https://www.ncbi.nlm.nih.gov/pubmed/?term=Sack%20DA%5BAuthor%5D&cauthor=true&cauthor_uid=12147489)., 2002, Isolation of Shigella dysenteriae type 1 and S. flexneri strains from surface waters in Bangladesh: comparative molecular analysis of environmental Shigella isolates versus clinical strains. Appl Environ Microbiol, 68, 3908-13.

3.Vargas, M., Gascon, J., Maria, T.J.D.A., Vila, J., 1999, Prevalence of Shigella Enterotoxins 1 and 2 among Shigella Strains Isolated from Patients with Traveler's Diarrhea. J Clin Microbiol. 37, 3608-3611
