# Supplementary material for: Epidemic and molecular characterization of fluoroquinolone-resistant Shigella dysenteriae 1 isolates from calves with diarrhea
Source: BMC Microbiol. 2021 Jan 6;21:6. doi: 10.1186/s12866-020-02050-9 (PMC7789508; doi:10.1186/s12866-020-02050-9)
Supplement: Supplementary file 3 — Additional file 3: Table S3. Primers for the detection of fluoroquinolone resistance–determining genes. [file 12866_2020_2050_MOESM3_ESM.docx]

Table S3. Primers for detection of fluoroquinolone resistance determinants genes.

| Target | Primer sequence (5’ to 3’) | Amplicon size (bp) | Reference |
| --- | --- | --- | --- |
| **PMQRs** | | | |
| *qnrA* | F: ATTTCTCACGCCAGGATTTG | 516 | Colobatiu et al.,2015 |
|  | R: GATCGGCAAAGGTTAGGTCA |  |  |
| *qnrB* | F: GATCGTGAAAGCCAGAAAGG | 476 | Colobatiu et al.,2015 |
|  | R: ACGATGCCTGGTAGTTGTCC |  |  |
| *qnrD* | F: CGAGATCAATTTACGGGGAATA | 656 | Cui et al.,2015 |
|  | R: AACAAGCTGAAGCGCCTG |  |  |
| *qnrS* | F: ACGACATTCGTCAACTGCAA | 417 | Colobatiu et al.,2015 |
|  | R: TAAATTGGCACCCTGTAGGC |  |  |
| *aac(6′)-Ib-cr* | F: CCCGCTTTCTCGTAGCA | 544 | Colobatiu et al.,2015 |
|  | R: TTAGGCATCACTGCGTCTTC |  |  |
| *qepA* | F: CGTGTTGCTGGAGTTCTTC | 403 | Colobatiu et al.,2015 |
|  | R: CTGCAGGTACTGCGTCATG |  |  |
| **QRDR** | | | |
| *gyrA* | F: TACACCGGTCAACATTGAGG | 648 | Hu et al.,2007 |
|  | R: TTAATGATTGCCGCCGTCGG |  |  |
| *gyrB* | F: TGAAATGACCCGCCGTAAAGG | 309 | Hu et al.,2007 |
|  | R: GCTGTGATAACGCAGTTTGTCCGGG |  |  |
| *parC* | F: GTACGTGATCATGGACCGTG | 531 | Hu et al.,2007 |
|  | R: TTCGGCTGGTCGATTAATGC |  |  |
| *parE* | F: ATGCGTGCGGCTAAAAAAGTG | 290 | Hu et al.,2007 |
|  | R: TCGTCGCTGTCAGGATCGATAC |  |  |

Reference

1. Colobatiu, L., Tabaran, A., Flonta, M., Oniga, O., Mirel, S., Mihaiu, M., 2015, First description of plasmid-mediated quinolone resistance determinants and β-lactamase encoding genes in non-typhoidal Salmonella isolated from humans, one companion animal and food in Romania. Gut Pathog. 7,16.

2. Cui, X., Wang, J., Yang, C., Liang, B., Ma, Q., Yi, S., Li, H., Liu, H., Li, P., Wu, Z., Xie, J., Jia, L., Hao, R., Wang, L., Hua, Y., Qiu, S., Song, H., 2015, Prevalence and antimicrobial resistance of Shigella flexneri serotype 2 variant in China. Front Microbiol. 6,435.

3. Hu, L.F., Li., J.B., Ye, Y., Li, X., 2007, Mutations in the GyrA subunit of DNA gyrase and the ParC subunit of topoisomerase IV in clinical strains of fluoroquinolone-resistant Shigella in Anhui, China. J. Microbiol.45,168-70.
